# Surface functionalisation of nanodiamonds for human neural stem cell adhesion and proliferation

**DOI:** 10.1038/s41598-017-07361-y

**Published:** 2017-08-04

**Authors:** Alice C. Taylor, Citlali Helenes González, Benjamin S. Miller, Robert J. Edgington, Patrizia Ferretti, Richard B. Jackman

**Affiliations:** 1 London Centre for Nanotechnology and Department of Electronic and Electrical Engineering, University College London, 17-19 Gordon Street, London, WC1H 0AH UK; 2 Stem Cell and Regenerative Medicine Section, UCL Great Ormond Street Institute of Child Health, University College London, 30 Guilford Street, London, WC1N 1EH UK

## Abstract

Biological systems interact with nanostructured materials on a sub–cellular level. These interactions may govern cell behaviour and the precise control of a nanomaterial's structure and surface chemistry allow for a high degree of tunability to be achieved. Cells are surrounded by an extra–cellular matrix with nano–topographical properties. Diamond based materials, and specifically nanostructured diamond has attracted much attention due to its extreme electrical and mechanical properties, chemical inertness and biocompatibility. Here the interaction of nanodiamond monolayers with human Neural Stem Cells (hNSCs) has been investigated. The effect of altering surface functionalisation of nanodiamonds on hNSC adhesion and proliferation has shown that confluent cellular attachment occurs on oxygen terminated nanodiamonds (O–NDs), but not on hydrogen terminated nanodiamonds (H–NDs). Analysis of H and O–NDs by Atomic Force Microscopy, contact angle measurements and protein adsorption suggests that differences in topography, wettability, surface charge and protein adsorption of these surfaces may underlie the difference in cellular adhesion of hNSCs reported here.

## Introduction

Nano–bio interfaces encompass kinetic, physiochemical and thermodynamic interactions between the surfaces of nanomaterials and numerous biological components including proteins, cell membranes and DNA^1^. Cells are surrounded by extracellular matrix in their natural environment^2^; nanoscale topography is observed on the extra–cellular matrix (ECM) surface. The understanding of these nano–cell interactions is essential if advances in knowledge about cell motility, morphology, proliferation and differentiation are to occur^3^. It has been hypothesised that nanostructured surfaces are able to mimic live tissue^4^ as they have similar physical properties to the naturally occurring ECM^5^. Therefore, interest into studying the interactions of cells with nanostructured materials is increasing. Ideally, nanomaterials will be designed with precise biological functionality in order to control cell behaviour via external cues. This could be achieved by modifying chemical and physical properties of nano–scale materials^6^. It has been demonstrated that cellular behaviour is manipulated by a variety of substrate factors including rigidity^7, 8^, surface charge^9, 10^, topography^11, 12^ and wettability^13, 14^. Focal adhesions are molecular assemblies in which regulatory signals and mechanical forces can be transmitted between the ECM and cells^15^. They are generally between 5–200 nm in size, and it has been shown that these adhesion sites are greatly influenced by complex mechanisms which occur at the nano– rather than micro–scale^16^.

Stem cells have vast potential as treatment and prevention tools in regenerative medicine. However, it is essential that methods are developed for introducing cells into foreign environments whereby natural cell behaviour is maintained^17^. Neural Stem Cells (NSCs) are able to proliferate, self–renew and differentiate into the three main cell types present in the central nervous system: neurons, astrocytes and oligodendrocytes^18^. Understanding the differentiation into these specific cells is vital for advances in the treatment of neurological diseases such as Parkinson’s^19^ and Alzheimer’s^20^ to be made^21^. In order to utilise the regenerative potential of stem cells in treating neurodegenerative diseases, the stem cell ‘niche’ must be found. The niche is the specific microenvironment in which stem cells naturally occur. The interaction of cells with this exterior niche environment influences stem cell fate^22^. In order to mimic this niche, nano–biomaterials are being precisely engineered to enable specific stem cell manipulation and interaction. Examples include but are not limited to: graphene and graphene foams^23, 24^, carbon nanotubes^25, 26^, and various other nanofibers^27–29^.

Diamond is considered to be a biocompatible material^30–34^; this along with the excellent electrical properties of diamond^35, 36^ make it an exciting material for electrically interfacing with neurons. Detonation nanodiamonds (DNDs) were first synthesised at the beginning of the 1960s^37^. Being, typically between 5–10 nm in diameter, these nanoparticles naturally aggregate into micro sized particles due to high Van der Waals (VdW) intermolecular forces. Developments in the dispersion of DNDs has enabled monolayers of DNDs to be produced attached to various substrates^38^. Neurons have been successfully grown on single crystal^39^, micro–crystalline^34, 40^ and nanocrystalline diamond (NCD) films^41^. Nanodiamonds (NDs) have been shown to promote neurite outgrowth from neurons^42^ and patterned neural networks have been created by culturing neurons on nanodiamond tracks^43^.

NSCs are more sensitive than neurons. They are extremely responsive to external stimuli, and can readily aggregate to form balls of neural cells known as neurospheres. Neurosphere formation is indicative of poor NSCs adhesion to the biomaterial^44^. The interaction of NSCs with diamond has been reported: ultra–nanocrystalline diamond has shown to be a promising biomaterial of choice for NSC adhesion and differentiation^45^, with tunable cell adhesion being observed^46^. Microcrystalline diamond has also shown to be a successful platform for neuronal induced differentiation from pluripotent stem cells^47^. The current authors previously reported that boron doped diamond successfully supports the adhesion and proliferation of human NSCs (hNSCs) with an increase in adhesion being observed with increasing nanostructuring^31^. Previous studies have explored the use of nanodiamond monolayers for supporting rodent neurons^42^ and NCD with rodent NSCs^45^, despite the thorough scientific content of these publications, the importance of using human cells *in vitro* has been demonstrated^48, 49^. In the current paper, the interaction between ND monolayers and human NSCs has been investigated for the first time. Specifically, the effect of altering ND surface functionalisation on hNSC adhesion and proliferation after 7 days *in vitro* (DIV) has been explored. It has been shown that NDs with oxygen containing groups on the surface allow for a significantly better hNSC attachment over hydrogen functionalisation. Contact angle measurements and protein adsorption experiments have enabled the development of a probable explanation as to why this difference is observed.

## Results

### Roughness measurements

**Figure 1:**
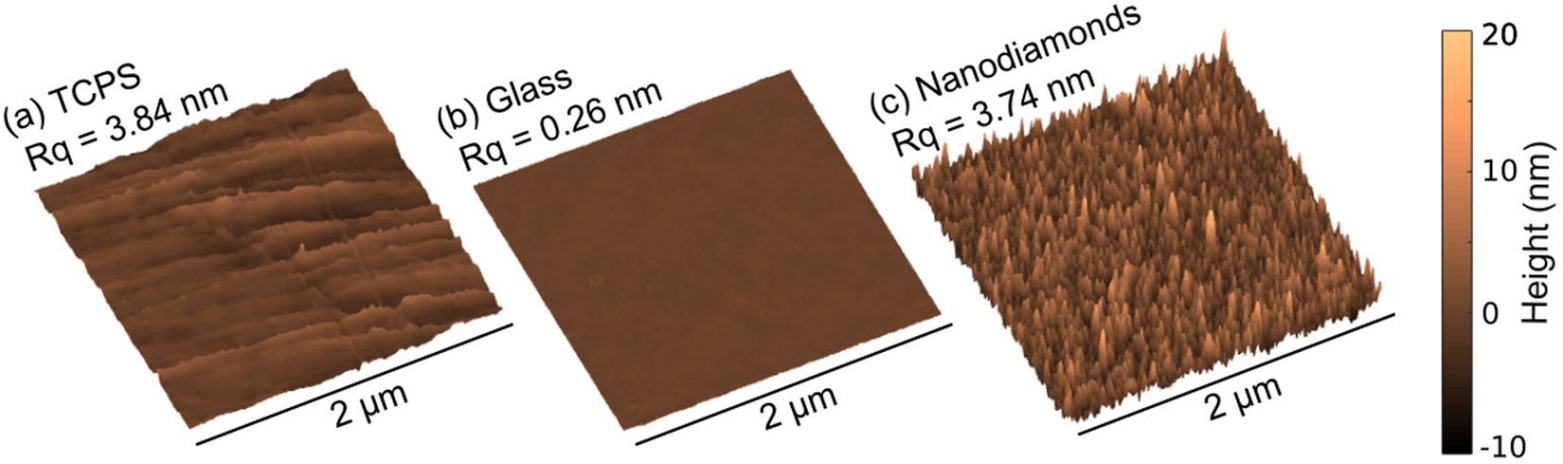
Atomic force microscopy (AFM) scan of 2 µm square (**a**) TCPS, (**b**) Glass and (**c**) Nanodiamonds. R_q_ = root mean square roughness.

AFM (Atomic Force Microscopy) topographical images of the three different types of substrate used throughout is shown in Fig. 1. The AFM data files were used to calculate root mean squared roughness (R_q_) measurements for each substrate. The roughness of the TCPS and ND monolayer were very similar (3.84 nm and 3.74 nm respectively), with the glass being much flatter at 0.26 nm. The AFM images also show that the NDs have adhered to the glass cover slips homogeneously in an apparent monolayer formation.

### XPS Measurements

**Figure 2:**
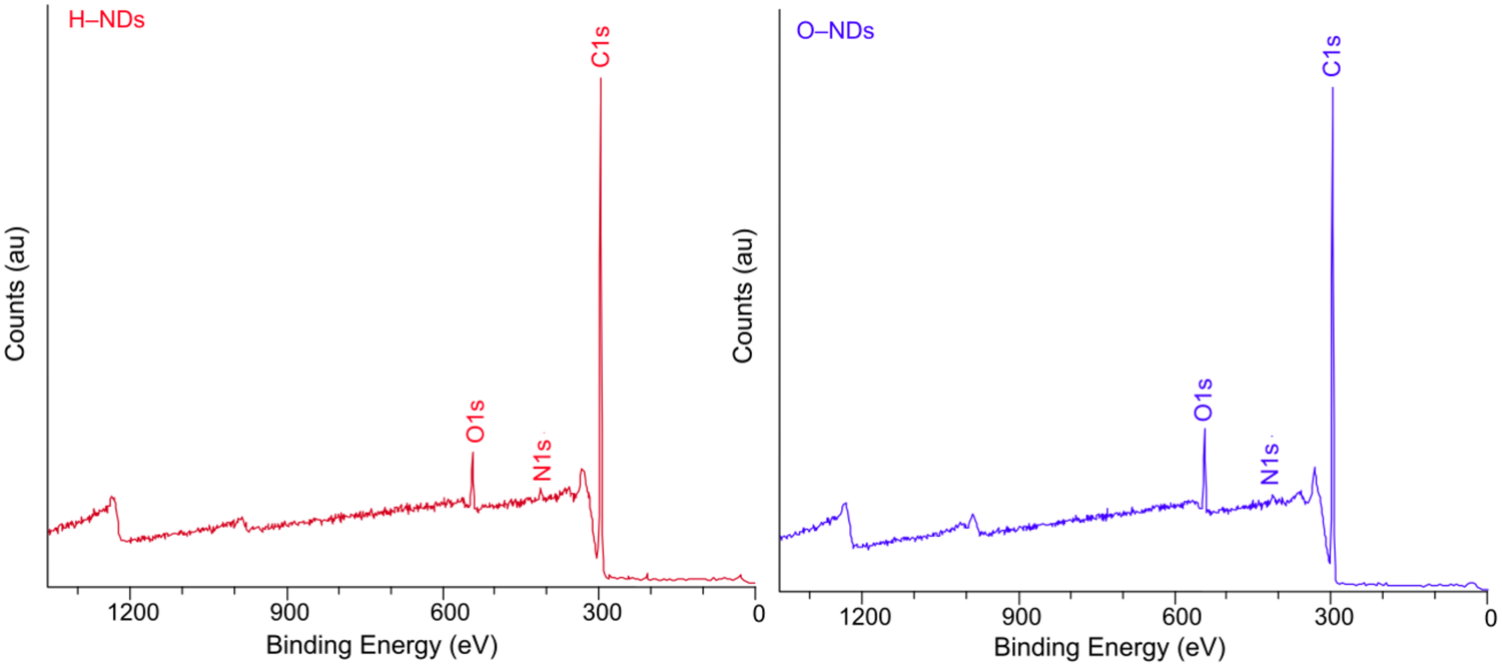
XPS spectra for (**a**) H–NDs and (**b**) O–NDs. Highlighted peaks are the C1s, O1s and N1s.

Figure 2 shows the XPS measurements for Hydrogen (a) and Oxygen (b) terminated nanodiamonds taken on supporting Indium tape. For both samples the nitrogen percentage composition is very similar (H–NDs 1.0% and O–NDs 0.9%), which suggest that the Nitrogen present is within the nanodiamond. The main difference in elemental composition between samples is the calculated difference in percentage attribution of the carbon and oxygen. H–NDs are 4.0% oxygen and 95.0% carbon, compared to O–NDs which are 6.8% oxygen and 92.2% carbon, this corresponds to a 70% increase in oxygen and a 3% reduction in carbon after O–termination.

### Optical images

**Figure 3:**
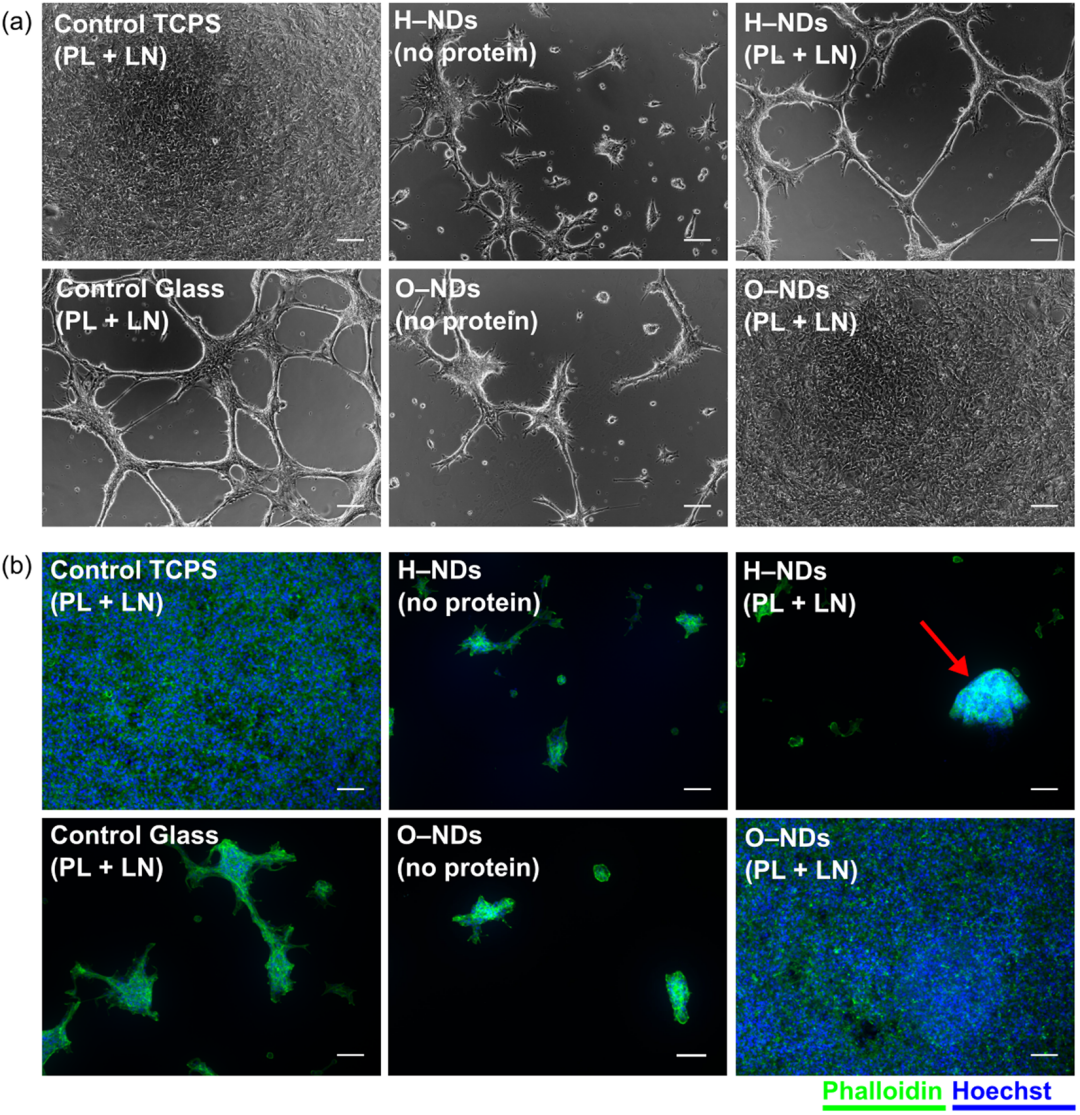
Micrographs of live hNSCs and cell stained for actin grown on different surfaces: (**a**) optical images of live hNSCs after 4 DIV (days *in vitro*) which have been cultured on Control TCPS, Control Glass H–NDs and O–NDs, with and without PL + LN (Poly-L-lysine + laminin) coating. (**b**) hNSCs fluorescently labelled using Alexa Fluor® 488 Phalloidin (green) to detect actin and Hoechst dye (blue) to visualize nuclei after 7 DIV on Control TCPS, Control Glass H–NDs and O–NDs, with and without PL + LN coating. The red arrow highlights a potential neurosphere on the H–NDs. Representative images for each substrate are shown. All scale bars are 100 µm.

Figure 3(a) shows optical images taken at 4 DIV of the hNSC culture. The optical images are useful to look at how the cells are adhering to each surface; it can be seen that by 7 days in culture hNSCs have become confluent (reached 100% coverage of seeded surface) on both the TCPS control and O–NDs, showing that these surfaces are ideal for hNSC attachment and their exapansion. In the glass control images, patchy, non–confluent adhesion of hNSCs to the surface is observed, indicating that the glass used herein is a non–ideal biomaterial for hNSC growth. The H–NDs appear to be the least suitable substrate for hNSC adhesion, with the lowest coverage of hNSCs observed. In fact, it appears that on glass, O–NDs without protein and H–NDs with and without protein, the hNSCs tend to adhere to each other rather than to the substrate forming clusters of cells that are indicative of formation of neurospheres (spherical aggregate of neural cells that grows in suspension).

### Fluorescently stained hNSCs

Phalloidin and Hoechst fluorescent dyes were used to selectively stain the F–actin of the microfilament in the cytoskeleton (green) and nuclei (blue) respectively in hNSCs after 7 DIV (days *in vitro*). F–actin is present in all types of eukaryotic cells, where it forms networks that support the mechanical structure, determine shape and facilitate movement of cells, enabling cell migration and division^50^. Figure 3(b) shows the fluorescent staining of hNSCs cultured on TCPS, glass and H– and O–NDs. It can be seen that both the TCPS control and O–NDs are highly suitable substrates for hNSC adhesion as a confluent cell layer is again observed, consistent with the optical images. In contrast, patchy adhesion is observed on both the glass control and H–NDs, with the presence of hNSC clusters indicating preferential cell–cell adhesion over cell–substrate adhesion, which usually leads to neurosphere formation. A potential neurosphere is observed in Fig. 3(b) H–NDs and has been indicated with a red arrow. The difference observed between micrographs of live cells or after fixation and actin labelling could be due to the several washing steps involved in the staining protocol, as cells which are loosely attached are more vulnerable to detachment.

### Cell count data

**Figure 4:**
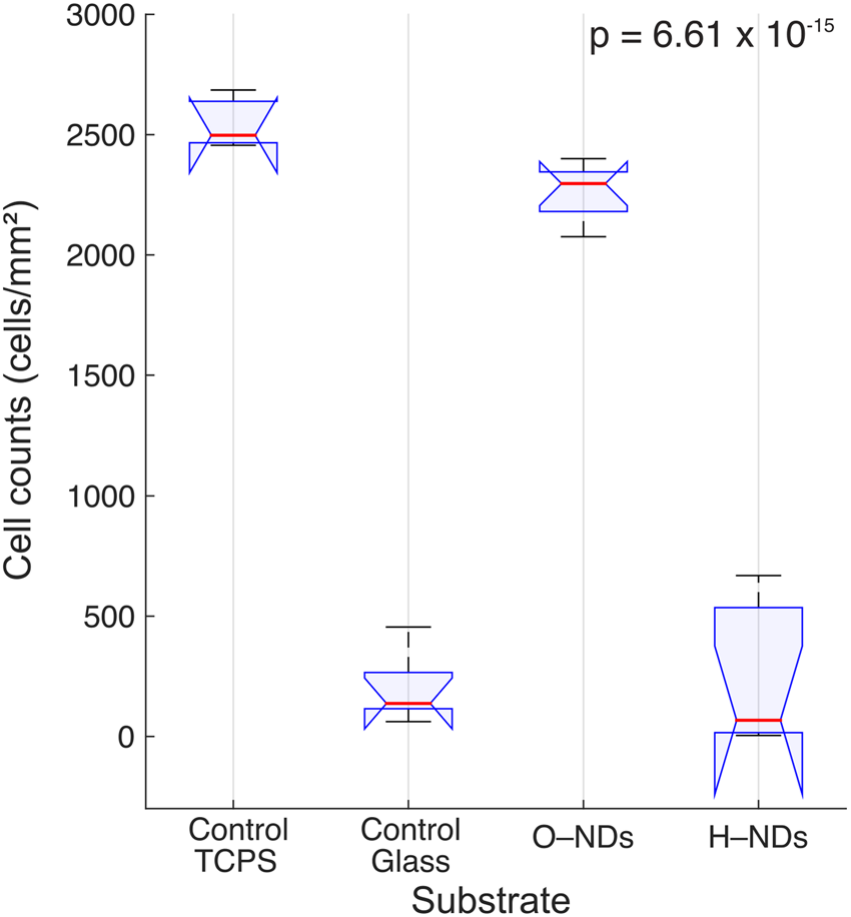
ANOVA analysis for hNSCs counted on Control TCPS, Control Glass, O–NDs and H–NDs (with protein coating) displayed as a boxplot; n = 6–8 images used for each substrate. Red horizontal lines represent the medians per substrate group in (cells/mm^2^), blue horizontal lines represent upper and lower quartiles, with blue notches signify a 95% confidence interval around the median. Black lines show the range of cell counts. Multicomparison analysis shows that Control TCPS and O–NDs are not statistically distinguishable, neither is Control Glass and H–NDs, however a statistical significance is observed between these two groups. A p–value of 6.61 × 10^−15^ has been calculated.

Cell count and subsequent ANOVA analysis (Fig. 4) was performed after 7 DIV. The highest cell count was observed on the TCPS (2546 ± 122 cells/mm^2^) followed by O–NDs with only slightly fewer cells (2265 ± 116 cells/mm^2^). A much lower number of cells was found on glass (198 ± 67 cells/mm^2^) and H–NDs (259 ± 173 cells/mm^2^).

### Contact Angle

**Figure 5:**
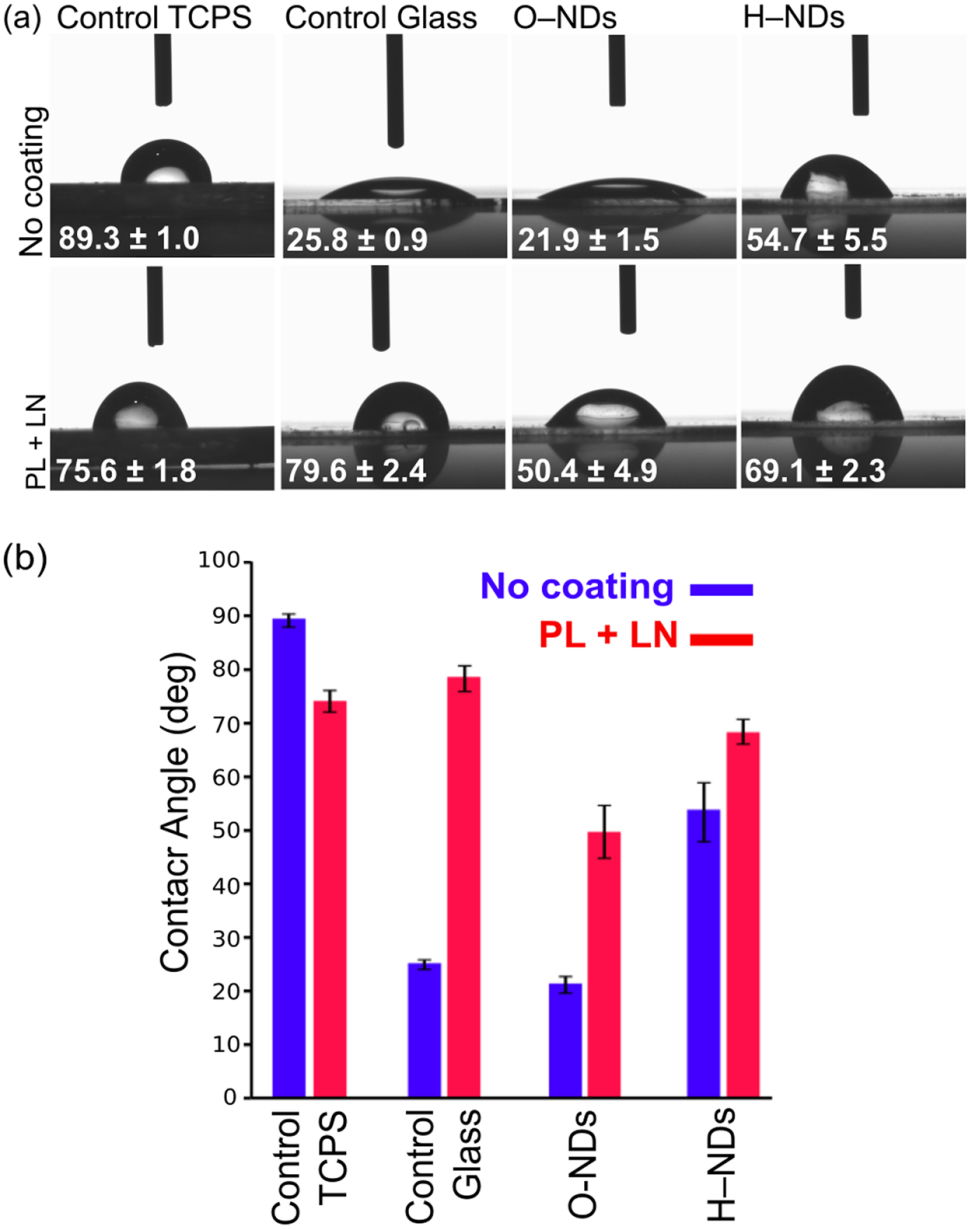
(**a**) Optical images showing contact angle values for 3 µl of DI water shown on (from left to right) Control TCPS, Control Glass, O–NDs and H–NDs. The top row is without any protein coating, and the bottom row is after (PL + LN) coating. (**b**) Shows the values obtained from triplicate drops, highlighting the difference with and without coating. Error bars show one standard deviation. ANOVA analysis (available in the supplementary material) of contact angle measurements showed that all 8 substrate types were statistically distinguishable, with a p value of 1.8238 × 10^−113^.

Contact angle measurements (Fig. 5) were performed on TCPS, glass, ONDs and HNDs, with and without protein coating. Three comparisons have been made, the first is between the four uncoated substrates, the second between the four protein coated substrates and the third between each substrate type with and without protein coating. Without coating the O–NDs produce the smallest contact angle of all substrates (21.9°), then glass (25.8°), then H–NDs (54.7°), with TCPS having the largest contact angle of (89.3°). A smaller contact angle is indicative of a more hydrophilic surface, and thus H–NDs are more hydrophobic than O–NDs, and TCPS is significantly more hydrophobic than the three other substrates. Poly-L-lysine and Laminin (PL + LN) coating increased the contact angle on all substrates except for the TCPS. The most hydrophobic substrate observed after coating was the glass (79.6°), but the contact angle of the coated TCPS (75.6°) and the (PL + LN) H–NDs (69.1°) was comparable. The contact angle of the (PL + LN) O–NDs is significantly less than the other three (50.4°). The largest increase in contact angle upon protein coating is observed on glass (+53.8°), then O–NDs (+28.5°) and then H–NDs (+14.4°) and the TCPS is the only substrate in which a decrease in contact angle is observed. ANOVA analysis (available in the supplementary material) was used to determine that the contact angle measurements for each substrate were statistically significant from each other with a p value of 1.8238 × 10^−113^.

### Bio–layer Interferometry

**Figure 6:**
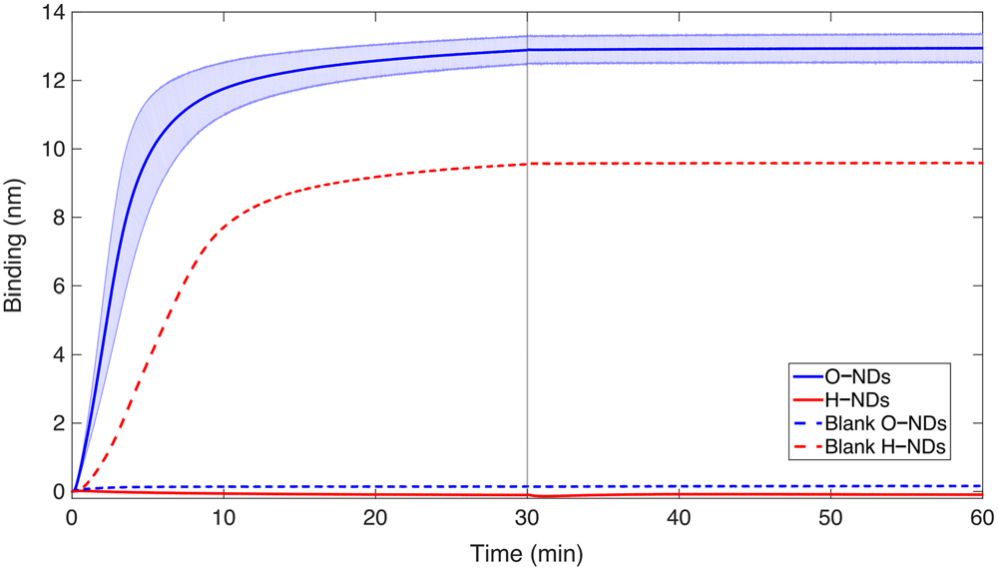
Bio–layer Interferometry data showing the difference in binding of H– and O–NDs to PL coated LN. The solid lines show the mean binding thickness of NDs onto the protein–coated sensor (O blue and H red). The pale blue and pale red block areas show the standard deviation from the mean. The dashed lines show the binding thickness of NDs (O blue and H red) of the blank sensors without any protein coating. The grey line at 30 minutes shows the transition of the biosensor from the ND coating solutions into DI water.

The binding of NDs with H– and O– surface functionalisation to sensors coated with (PL + LN) showed O–NDs to adhere considerably better than H–NDs. This is shown in (Fig. 6); the red solid line remains at 0 nm of binding for the duration of the association, showing that the H–NDs are not binding to the protein coated sensor at all. The opposite can be seen for the O–NDs, (blue line) which clearly shows fast binding. After 10 minutes a binding thickness of around 12 nm can be seen. The size of the O–NDs is approximately between 5–10 nm and so this observed thickness indicates NDs are completely covering the sensor and few aggregates are present. After 30 minutes the sensors are placed in DI water. The lack of any dissociation shows the stability of these O–NDs bound to the (PL + LN). The dashed lines in (Fig. 6) show the binding of H– and O–NDs to blank sensors; without protein coating. Here the opposite effect is observed; the H–NDs adsorb onto the sensor and the O–NDs do not. The binding rate is similar, and the stability of the ND–sensor interaction remains.

## Discussion

The adhesion and cell count of hNSCs after 7 DIV on (PL + LN) coated O–NDs has been shown to be as high as when cultured on TCPS. This can be seen in optical and fluorescently labelled hNSCs (Fig. 3a,b) and via cell counting data (Fig. 4). The adhesion and cell counts for hNSCs cultured on both glass and H–NDs is significantly lower. The surface functionalisation of NDs is therefore paramount in controlling cell behaviour.

Surface properties including wettability and roughness play a very important role in manipulating cellular adhesion. Modifications to surface properties can lead to improved biocompatibility^51, 52^, and it is very important that optimal conditions for specific cellular adhesion are realised. Nanotopography of surfaces greatly influences cellular adhesion, spreading^53^ and fate^54^. Nanometer rough scaffolds have shown to promote the adhesion and proliferation of nerve cells due to their high surface area^4^. Very similar roughness values for TCPS and ND monolayers are observed (Fig. 1), with glass being much smoother (TCPS: R_q_ = 3.84 nm, NDs: R_q_ = 3.74 nm, glass = 0.26 nm). O–NDs provide an excellent surface for hNSCs adhere to. The naturally occurring ECM of the stem cell niche is enriched with nanotopographical cues that manipulate cell fate and function^55^. It is hypothesised that the nano–scale roughness of ND monolayers is comparable to the ECM in the stem cell niche, which suggests why excellent hNSC adhesion is observed. The increase in surface area upon nanostructuring leads to more available sites for focal adhesion to occur^56^. It has previously been demonstrated that the focal adhesion activation of human fetal osteoblastic cells were significantly enhanced on PLLA plastic with approx. 5 nm R_q_ roughness^57^, comparable to ND monolayers (R_q_ = 3.74 nm) and TCPS (R_q_ = 3.84 nm). It is therefore suggested that the nano–scale roughness of NDs and TCPS promotes the formation of focal adhesions, as observed by actin staining, and thus enhances hNSC attachment.

Many chemical properties of a material including composition and hydrophilicity^58^ affect cellular adhesion. Typically, hydrophilic surfaces enable better adhesion despite having a lower affinity for protein adsorption than hydrophobic surfaces^59^. Contact angle results herein show the contact angle of O–NDs to be much less than TCPS, glass and H–NDs, both with and without PL + LN coating (Fig. 5). This increase in hydrophilicity of the O– functionalisation has transformed the surface from one that is not suited to hNSC adhesion, into one that is. The similarity in contact angle after coating with (PL + LN) for TCPS, glass and H–NDs suggests that the proteins are adhering to the surfaces similarly. The significant difference observed between O–NDs and the other three substrates with (PL + LN) coating, suggests that oxygen–containing groups on the surface of the NDs are interacting differently with the proteins. H– and O–NDs have positive and negative surface charges respectively^60^. PL has many available amino groups, which are hydrophilic in nature and are positively charged^61^. It is hypothesised that the negatively charged O–NDs are electrostatically attracting the positively charged PL, resulting in a stronger PL binding with better coverage. This increased coverage of positively charged PL subsequently promotes uniform coating of LN, which is negatively charged^62^. LN enhances adhesion and growth of NSCs^63^, therefore uniform coverage results in a more confluent attachment of cells. The surface of TCPS is also negatively charged^64^. hNSC adhesion is high on (PL + LN) coated TCPS, this may also be a result of the electrostatic interactions between the negatively charged surface and the positively charged PL. It is suggested that the positively charged surface of the H–NDs electrostatically repulses the PL inducing a non–uniform coating of LN, which results in the reduced and patchy cell adhesion observed.

**Figure 7:**
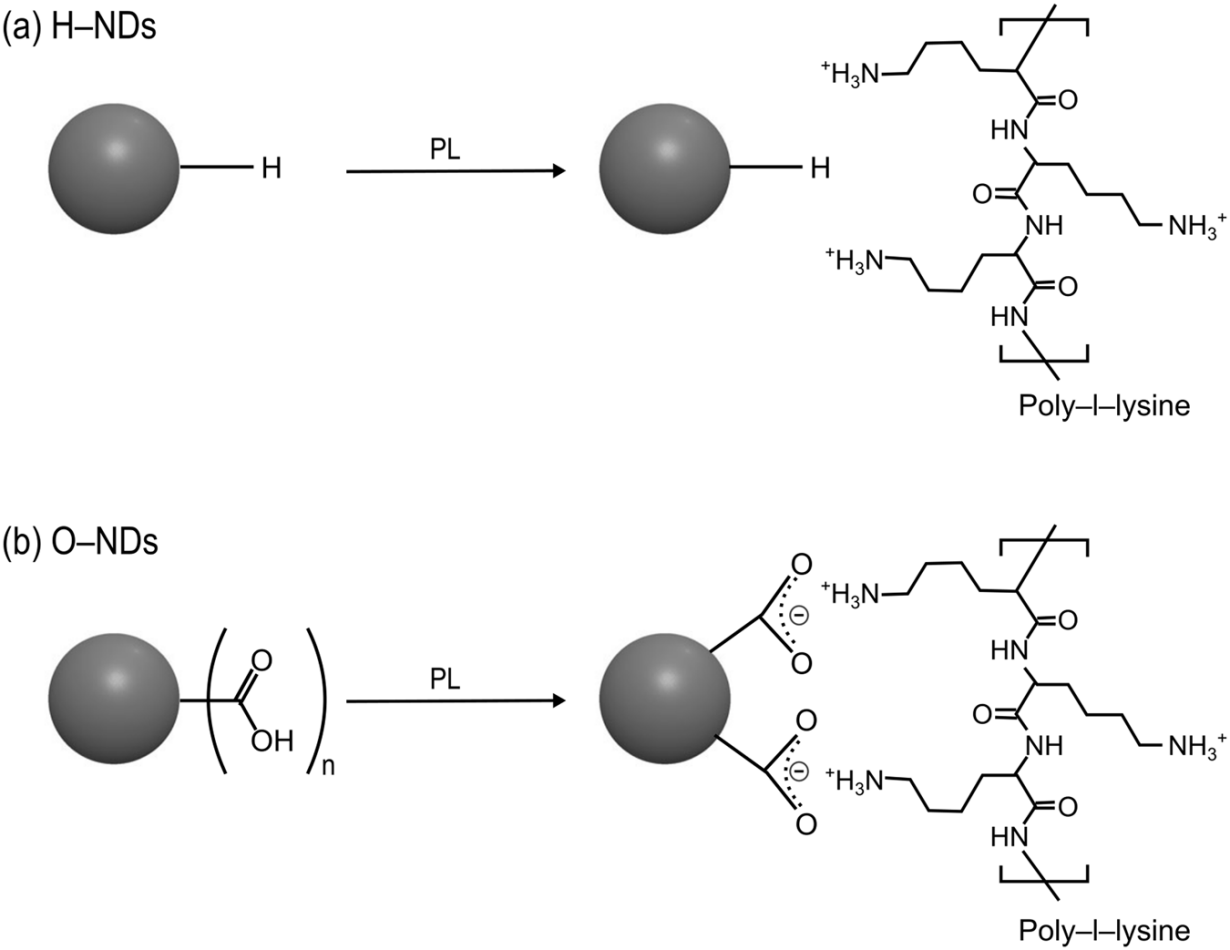
Schematic outlining the bonding of H– and O–NDs with poly–L–lysine. (**a**) Shows the lack of binding interactions occurring between H–NDs and PL. (**b**) Surface modification of O–NDs with PL, resonant negatively charged structures on the surface of the O–ND conjugate with the positively charged PL.

BLI results show that O–NDs bind readily to PL coated LN, compared to H–NDs which showed no binding. Surface charge of NDs has shown to have a substantial affect on protein adsorption^60^. Contact angle results suggest that electrostatic interactions of the H– and O–NDs are responsible differences in protein adsorption. This explains why the negative O–ND surface binds readily to the positively charged PL, whereas the positive H–NDs do not. Suggested electrostatic interactions occurring between PL and H– and O–NDs can be seen in (Fig. 7) (adapted from ref. 65). It is believed that the positive NH_3_^+^ groups on the PL are non–covalently bonding to the stable negatively charged resonant carboxyl groups present on the surface of the O–NDs, in contrast to the lack of interaction occurring between the hydrogen groups present on the surface of the H–NDs.

hNSCs typically require surfaces coated with large ECM proteins, such as LN, to maximize integrin-mediated adhesion^66^. Evidence suggests that negatively charged oligosaccharides groups present in LN are responsible for protein–protein bonds^67^. This has been confirmed using BLI, where binding between LN and PL is observed, remaining stable after placing the biosensor in DI water. The stability of the binding between O–NDs and PL suggests that the electrostatic interactions occurring are strong. It is hypothesised that the monolayer coverage of NDs and the protein adsorption affinity of O–NDs is facilitating the uniform coverage of PL. The lack in binding of H–NDs with PL suggests that the coating procedure is resulting in uneven PL coverage. This supports hNSC adhesion observations, where minimal attachment occurs.

Contact angle and protein adsorption results reported; suggest why excellent adhesion and high cell counts of hNSCs cultured on O–NDs is observed

In conclusion, good adhesion of cells onto biomaterials is paramount in enabling the proliferation and maintaining cellular functions^24^. The dangling sp^2^ bonds on the surface of NDs enable precise control over termination^68^. It has been shown that the presence of oxygen containing groups on the surface of NDs has increased hNSC adhesion compared to NDs with hydrogen–termination. A number of factors have been suggested as to why hNSCs have such pronounced adhesion to O–NDs but not to H–NDs. These include: topography, wettability, surface charge and protein adsorption, and it has been hypothesised that hNSC adhesion results from a combination of all of these factors. Modifying biomaterial surfaces in order to create nano–roughness has previously resulted in an increase in cellular adhesion on plastic with an R_q_ of 5 nm^57^, the R_q_ value for ND monolayers is approx. 4 nm. This similarity in nano–roughness is proposed to be ideal for hNSC adhesion. Contact angle values for O–NDs were much lower than TCPS, glass and H–NDs, with and without protein coating. This increase in hydrophilicity is also responsible for the increase in hNSC attachment.

hNSCs are typically grown on surfaces coated with laminin, a large ECM protein. The large size results in several integrin binding motives per protein, which allows engagement with numerous integrin receptors on the hNSC surface^66^, and therefore an increase in focal adhesion sites. The O–NDs have shown to promote adhesion via an increase in protein adsorption, and it is hypothesised that this increase in LN and PL coverage has led to the development of more available focal adhesion sites. Typically, an increase in hydrophilicity corresponds to a decrease in protein adsorption^59^. Despite this, O–NDs enable the simultaneous increase in hydrophilicity and protein adsorption affinity.

These results suggest that modifications to ND functionalisation provides extensive potential for discovering and exploiting NDs as a bio–material. It is proposed that the interactions of cells with NDs can be controlled via precisely tuning surface functionalisation. The simple sonication process in which objects are coated, promotes the use of NDs as an *in vivo* biomaterial. Specifically, it is suggested that O–NDs could be a promising material of choice for the coating of neurological implants in which NSC adhesion is paramount, further *in vivo* testing is required.

## Methods

### Chemicals

Monodispersed detonation nanodiamonds (DNDs) (5–10 nm) were used throughout (New Metals & Chemicals Corporation, Tokyo, Japan). This form of DND has been subjected to a deagglomeration process utilising wet ball milling with zirconia^69^.

### Nanodiamond monolayer coatings

Nanodiamond monolayer coatings are obtained by ultrasonicating substrates in the ND solution (0.05 g/L of NDs) for 10 minutes (excess time). Glass (Cover glass, Menzel–Gläser, Thermo Scientific, UK) was used throughout as the substrate for ND attachment as the transparency allowed for easy optical and fluorescent imaging. Prior to seeding substrates were degreased in acetone, isopropyl alcohol (IPA) and then deionised (DI) water (each for 5 minutes sonication), to remove any residues.

### Hydrogen termination

DNDs were dried by evaporating off excess water at 80 C for 30 minutes. Hydrogen functionalization of these DNDs was achieved using a hydrogen anneal process. A custom–made chamber was used to heat samples to 600 C in 25 Torr of hydrogen for 5 hours and allowed to cool in hydrogen. H–NDs were then re–suspended in DI water (0.05 g/L) and subjected to ultra-high power sonication using a VCX500 Vibra-cell sonicator with the cup horn accessory (100% amplitude, 3:2 duty cycle, water cooled and temperature controlled to be <30 °C, 5 hours) to fully disperse the NDs.

### Oxygen termination

Oxygen functionalization of the NDs was achieved using an ozone treatment on H–ND monolayers. A custom built chamber was used in conjunction with a commercially available ozone generation unit (Ozonia TOGC2–100201). Here, samples were subjected to ozone flow at a pressure of 50 mbar, at 200 C for 1 hour. After which the sample was allowed to cool in ozone before being removed.

### Atomic Force Microscopy

Atomic force microscopy (AFM) measurements were carried out using a Veeco Dimension V instrument with aluminum-coated silicon AFM probes (resonant frequency 190 kHz). The system was operated in tapping mode with a VT-103-3K acoustic/vibration isolation system and a VT-102 vibration isolation table at room temperature in air. AFM was performed on Tissue Culture Polystyrene (TCPS) (Costar 3527, Corning, 24 well plate), glass H–ND monolayers. Scan sizes of 2 μm were taken and root mean square of the roughness (Rq) was calculated using Nanoscope Software 6.1.3. AFM Images were post processed with a median filter (3 × 3 kernel) using MATLAB 2012a software to remove noise and measurement artifacts.

### X-ray Photoelectron Spectroscopy

Surface chemistry of the nanodiamond samples was characterised by X-ray photoelectron spectroscopy (XPS) performed using a Thermo K–alpha instrument, a monochromated Al K_a_(1486.6 eV) radiation source being used, alongside an internal flood gun to reduce charging. Data was processed and analysed using CasaXPS (version 2.3.12, Casa Software Ltd).

### Contact Angle Measurements

Contact angle measurements were taking using a Krűss Drop Shape Analysis system (DSA 10 MK2), values were calculated using Drop Shape Analysis software. Contact angles of the DI water droplet were measured immediately, with a constant volume of 3 µl used for all samples. Twelve repeats were done for each substrate; statistical analysis was performed using the one–way analysis of variance (ANOVA) test. MATLAB 2015a software was used to perform ANOVA analysis on the contact angle measurements, multicompare analysis is used to determine whether a statistically significant difference is observed.

### Human Neural Stem cell Isolation and Culture

All procedures involving human tissue were carried out in accordance with the UKs Human Tissue Act 2006. The hNSCs were isolated and expanded according to the protocol described previously^70, 71^. Briefly, whole brains from human embryos at Carnegie stage 17 (approx. 41 days) from consenting patients were provided by a tissue bank under ethical approval (NRES Committee London – Fulham, UK) were collected through the Human Developmental Biology Resource (HDBR, http://hdbr.org) and dissected in cold Neurobasal medium (Gibco). After complete removal of the meninges and blood vessels, the tissue was chopped into smaller pieces and digested in Accutase (Gibco) solution at 37 °C for 30 minutes with occasional trituration to obtain single cell suspension. Cells were then centrifuged and re-suspended in growth medium composed of DMEM/F12 with Glutamax ™ supplemented with 1% (v/v) Penicillin/Streptomycin, 1% (v/v) 100× N2 supplement, 2% (v/v) 50× B27 supplement (all Gibco), 20 ng/ml human recombinant FGF2, 20 ng/ml human recombinant EGF (both Peprotech), 50 μg/ml BSA fraction V and 5 μg/ml Heparin (both Sigma). Cells were plated on laminin (10 µg/ml, Sigma) – coated dishes and grown for 7 days *in vitro* with the media changed every 2 days to remove any dead cells or debris. To eliminate neurons from the primary cultures and get a homogenous culture of neural stem cells, the cells were first transferred onto 0.1% (w/v) bovine gelatine (Sigma)–coated dishes for 7 days to form neurospheres, which were then re–plated onto laminin–coated dishes for further expansion. For routine expansion and further experiments, cells were grown in growth media supplemented with laminin instead of coating the dishes. Passages up to 30 were used for all experiments.

### Cell attachment and morphology assay

After nanodiamond monolayer seeding and functionalisation, substrates were placed in 24 well tissue culture plates, sterilized for 30 minutes in 70% ethanol and washed three times in phosphate buffer saline (PBS). Prior to cell culture, some substrates were coated with poly–L–lysine (PL) (Sigma 1 mg/ml) and laminin (LN) (Sigma, L2020, 1 mg/ml). PL was diluted to 0.1 mg/ml in PBS. Sufficient solution was then placed into each well to ensure even coating of surface for 1 hour at room temperature. Substrates were washed 3 times with PBS and left in the hood over night or until dry. After PL coating, LN solution was diluted to 10 μg/ml in PBS. Sufficient solution was added to each well to ensure even coating of surface and incubated for one hour at 37 °C. Excess LN was then removed and used without washing. hNSCs were then plated at a density of 2 × 10^4^ cells/cm^2^ and were grown in humidified incubators at 37 °C with 5% CO_2_. For actin staining hNSCs were fixed after 7 *days in vitro* (DIV*)* in 4% (w/v) paraformaldehyde in PBS (pH 7.4) for 15 minutes and washed three times with PBS. Substrates were then incubated in blocking solution (10% fetal bovine serum, 3% bovine serum albumin in PBS) with 0.2% Triton-X 100 for permeablization for one hour at room temperature. Cells were stained with Phalloidin conjugated with Alexa Fluor® 488 diluted in blocking buffer (Invitrogen, 5 U/ml) together with Hoechst 33258 (2 µg/ml) to counterstain nuclei for 1 hour at room temperature. After 3 washes with PBS to remove excess dye the samples were imaged using an inverted microscope Olympus IX71 (Carl Zeiss, Jena, Germany) equipped with a Hamamatsu ORCA-R2 digital camera (Hamamatsu Corp., Bridgewater, NJ). Images were processed using Image J and Fiji^72^. Cell counts were obtained for the Hoechst stained images in Matlab R2015a by applying an intensity threshold to the stained images and then using an edge finding transform to find the number of labeled nuclei per image, which was then scaled up accordingly (at least six images at 10x magnification for one sample per substrate). Statistical analysis was performed using the one–way analysis of variance (ANOVA) test. MATLAB 2015a software was used to perform ANOVA analysis on the cell count data, with multicompare analysis being used to determine whether a statistically significant difference in cell counts was observed.

### Bio–layer Interferometry

Bio–layer Interferometry (BLI) was performed using a (ForteBio Octet ^®^ RED96) to observe the adsorption of NDs onto proteins.

### Preparation of Laminin and Poly–l–lysine

Laminin (LN) (Sigma, L2020, 1 mg/ml) was diluted to 25 µg/ml in 10 mM sodium acetate buffer pH 4.0. Previous pH scouting experiments showed pH4 to achieve optimal LN immobilization. Poly-L-lysine (PL) (Sigma 1 mg/ml) was diluted to 0.1 mg/ml in PBS.

### Immobilization of Laminin via amine coupling on AR2G biosensors

**Figure 8:**
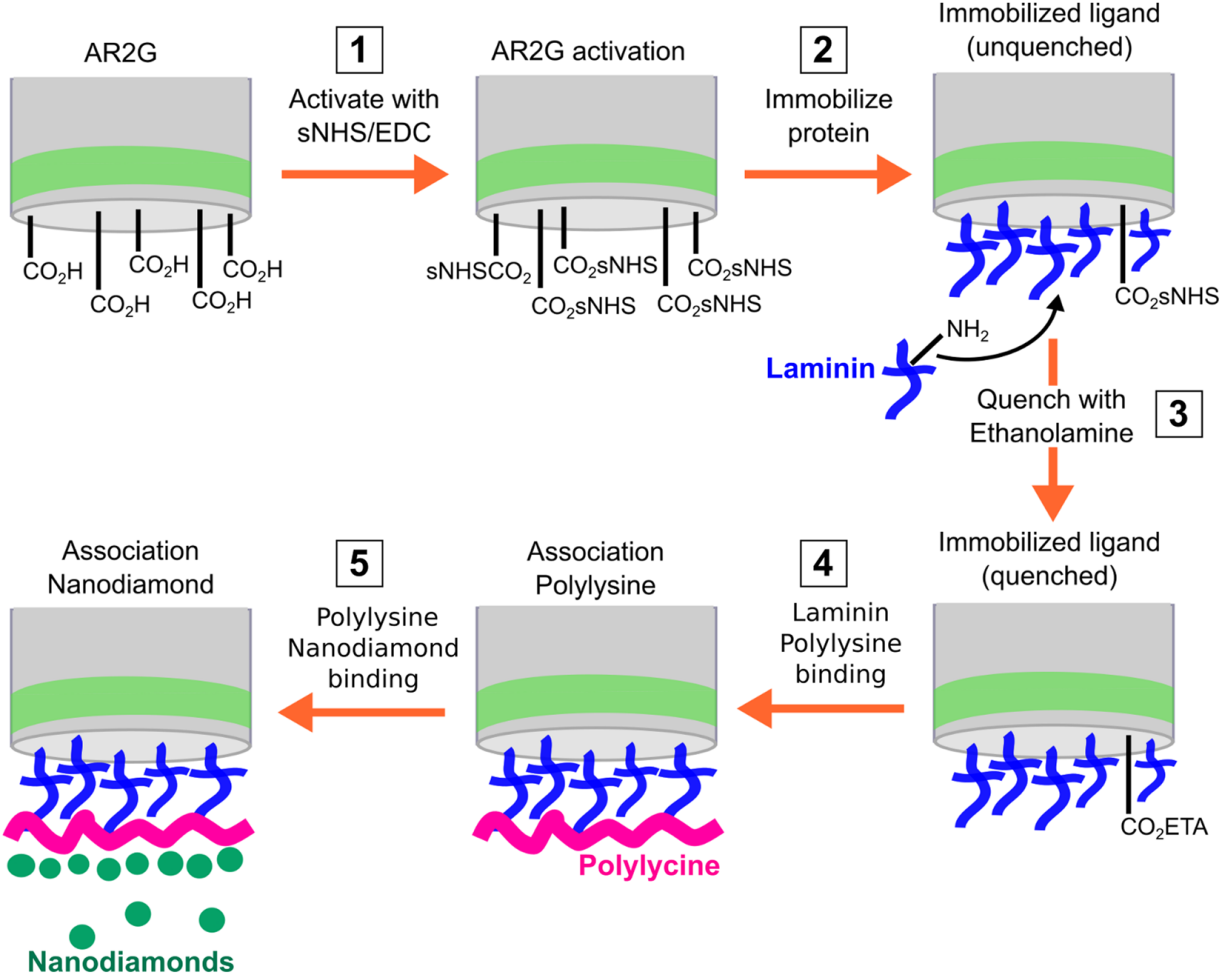
Schematic outlining the covalent immobilization process of laminin onto AR2G coated biosensors, and subsequent binding of poly-L-lysine and nanodiamonds. Step 1: EDC catalysis activates carboxylic acid coated sensor by facilitating the reaction with sNHS, resulting in a more reactive carboxylic acid site. Step 2: LN is covalently immobilized onto the sensor. The covalent bond is formed between a reactive amine group on the laminin and the carboxyl–functionalized surface of the biosensor. Step 3: ethanolamine is used to quench all remaining carboxylic activated groups on the sensor head to prohibit non–specific binding. Step 4: poly-L-lysine is bound to the laminin coated sensor, the thickness of this is measured using Bio–layer Interferometry. Step 5: nanodiamonds are bound to the poly-L-lysine, the binding thickness is also measured using Bio–layer Interferometry.

Amine Reactive 2nd Generation (AR2G) biosensors are used to immobilize LN onto the end of an optical fibre. The immobilization is achieved via a standard amide bond formation via EDC catalysis, which results in a covalent bond between the carboxylic acid terminated biosensor and a reactive amine on the LN. The biosensors were pre–hydrated in DI water for 10 minutes and then the AR2G surface is activated by reaction with 20 mM EDC (1-Ethyl-3-[3-dimethylaminopropyl] carbodiimide hydrochloride) and 10 mM s-NHS (N-hydroxysulfosuccinimide) in DI water for 5 minutes. LN (25 µg/ml, pH 4) is immobilized onto the surface for 15 minutes, which results in covalently bonding between the activated AR2G and amine groups on the LN. The immobilization was performed at 30 °C, and agitation speeds of 1000 rpm were used. The activated carboxylic groups are then quenched in 1 M ethanolamine for 5 minutes. A schematic of the immobilization can be seen in Fig. 8. Experimental definition and execution was performed using Data Acquisition software 7.1.0.92 (ForteBio).

### Association of Polylysine and Nanodiamonds

After activation of AR2G and immobilization of LN, the biosensors are buffered in PBS for 180 s to equilibrate. Sensors are then immersed in PL (0.1 mg/ml in PBS) for 30 minutes. Equilibration in DI water for 300 s showed minimal PL dissociation. The subsequent association of NDs (0.5 mg/ml) with both H– and O–functionalisation onto the PL was investigated for 30 minutes. Following association, dissociation in DI water was performed to gauge stability of adsorbed NDs. Both ND types were tested in triplicate.

### Association of NDs onto non–functionalised biosensors

The association of H– and O–NDs (0.5 mg/ml) onto quenched sensors (without LN and PL) was simultaneously investigated as a control.

## Electronic supplementary material

Supplementary information
