# Supplementary material for: Surface functionalisation of nanodiamonds for human neural stem cell adhesion and proliferation
Source: Sci Rep. 2017 Aug 4;7:7307. doi: 10.1038/s41598-017-07361-y (PMC5544760; doi:10.1038/s41598-017-07361-y)
Supplement: Supplementary file 1 — Supplementary information [file 41598_2017_7361_MOESM1_ESM.pdf]

Surface functionalisation of nanodiamonds for human neural stem cell adhesion and proliferation

Alice C. Taylor, Citlali Helenes González, Benjamin Miller, Robert J. Edgington, Patrizia Ferretti, Richard B. Jackman

Supplementary material

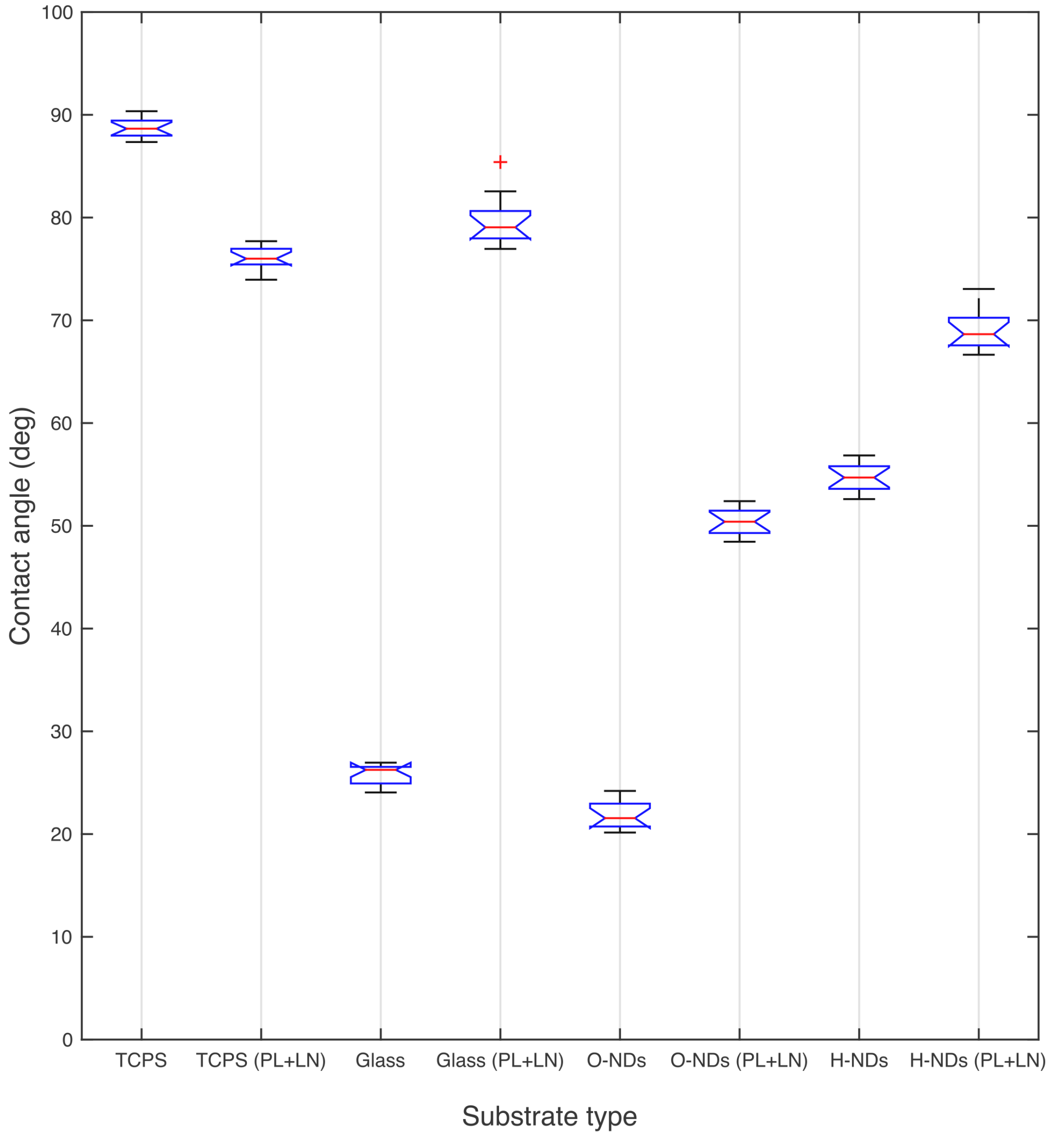

Supplementary Figure 1: ANOVA analysis for contact angle data on Control TCPS, Control Glass, O–NDs and H–NDs with and without polylysine and laminin coating, displayed as a boxplot, for (n=12). Red horizontal lines represent the medians per substrate group in degrees, blue horizontal lines represent upper and lower quartiles, with blue notches signify a 95% confidence interval around the median. Black lines show the range of cell counts. Multicompare analysis shows that all substrates are statistically different with a p–value of 1.8238 x 10-113 has been calculated.
